# Mortality Salience, System Justification, and Candidate Evaluations in the 2012 U.S. Presidential Election

**DOI:** 10.1371/journal.pone.0150556

**Published:** 2016-03-16

**Authors:** Joanna Sterling, John T. Jost, Patrick E. Shrout

**Affiliations:** Department of Psychology, New York University, New York, New York, United States of America; University of Amsterdam, NETHERLANDS

## Abstract

Experiments conducted during the 2004 and 2008 U.S. presidential elections suggested that mortality salience primes increased support for President George W. Bush and Senator John McCain, respectively. Some interpreted these results as reflecting “conservative shift” following exposure to threat, whereas others emphasized preferences for “charismatic” leadership following exposure to death primes. To assess both hypotheses in the context of a new election cycle featuring a liberal incumbent who was considered to be charismatic, we conducted four experiments shortly before the 2012 election involving President Barack Obama and Governor Mitt Romney. Contrary to earlier studies, there was little evidence that mortality salience, either by itself or in interaction with political orientation, affected overall candidate ratings or voting intentions. However, a significant interaction between mortality salience and system justification in some studies indicated a more circumscribed effect. The failure to “replicate” previous results in the context of this election may be attributable to disagreement among participants as to which of the candidates better represented the societal status quo.

## Introduction

For nearly a century, social theorists have struggled to understand how periods of high social, economic, and political threat influence public opinion (e.g., [1–5]). Over the last three U.S. presidential election cycles, threatening circumstances have been highly salient. Following the events of September 11, 2001, 85% of Americans stated that they were very or somewhat concerned about another attack, and 68% feared being personally affected by terrorism ([6], surveys collected between October 2001 and March 2002). As the election of 2004 approached, reminders of the terrorist attacks flooded the media, as did stories of spreading insurgency in Iraq. Shortly before the 2008 election, Lehman Brothers filed for bankruptcy—signaling the beginning of the most significant economic crisis to affect the U.S. since the Great Depression. Just a couple of weeks before the 2012 election, Hurricane Sandy, which was described as a “once-in-a-century” storm, destroyed a sizeable portion of the East Coast. More recently, in the days leading up to the mid-term 2014 elections, media coverage of ISIS (Islamic State of Iraq and Syria, a self-described caliphate violently imposing their interpretation of Sharia law on various regions of the Middle East) and the Ebola virus (most severely affecting Guinea, Sierra Leon, and Liberia, with a few cases surfacing in Western countries) dominated the airwaves. It seems clear that the question of how threat influences electoral politics should be of cardinal interest to social scientists.

Social psychological research has confirmed empirically that both large- and small-scale threats can, and sometimes do, influence political attitudes and behaviors [7–12]. One prominent finding observed both in the U.S. and abroad has been a shift towards conservative political ideologies, policies, and candidates following a threatening event, such as a terrorist attack [13–15]. “Conservative shifts” are not unique to large-scale threats—they have been documented with small-scale threats, such as being reminded of one’s own mortality in controlled experimental settings [16–18]. Based on the results of an extensive meta-analytic review of 88 studies from 12 different countries, Jost, Glaser, Kruglanski, and Sulloway proposed that conservative ideology may be “especially appealing to those who are situationally or dispositionally prone to experience fear or to find uncertainty aversive” ([19], p. 352).

In 2004 and 2008, a number of studies suggested that participants were indeed more likely to support more conservative presidential candidates (Bush rather than Kerry in 2004; McCain rather than Obama in 2008) when they were reminded of their own mortality [9, 20, 21]. One article suggested that this general tendency could be overcome by priming the concept of compassion [21]. In these studies, however, the mechanisms responsible for these effects remained unclear. Although Bush and McCain were more conservative than their opponents, they also had other attributes that might have been made more attractive by the mortality salience manipulation. These may include perceived charisma as well as an emphasis on specific conservative or authoritarian themes such as military hawkishness and the rhetoric of American superiority. Because these mortality salience experiments lacked measures that would enable one to distinguish among these various explanations, and the attributes were themselves correlated, it was not possible to establish which mechanisms drove the effects of mortality salience.

We revisit this issue and investigate competing predictions from the existing literature regarding how mortality salience might influence evaluations of presidential candidates in an electoral context in which political conservatism was not linked to incumbency status or an advantage in terms of perceived charisma. With a liberal incumbent, it is at least conceivable that the political climate in 2012 would be more conducive to the presence of a liberal shift in response to threat, in comparison with the 2004 and 2008 electoral environments. At the same time, it is possible that Obama’s mixed racial heritage might have limited the extent to which his appeal, even as an incumbent and a relatively charismatic candidate, would be heightened under mortality salience. In the present research program, we tested four contrasting predictions derived from system justification and terror management perspectives in four mortality salience experiments with experimental procedures that borrowed heavily from procedures used by terror management researchers to study the 2004 election.

### System Justification Theory

System justification theory (SJT) posits that, all other things being equal, individuals are motivated to perceive existing social, economic, and political arrangements as legitimate, fair, and just [22, 23]. These cognitive-motivational processes are theorized to be active for those whom the system advantages and, in some cases at least, for those whom the system places at a disadvantage. While all individuals possess system-justifying tendencies to some extent, SJT notes that both situational and dispositional factors can strengthen or weaken this motivation. Specifically, SJT predicts a stronger tendency to defend, justify, and bolster the societal status quo when an individual is confronted with existential or epistemic threats as well as threats to the legitimacy of the social, economic, or political system. Importantly, system justification tendencies are associated with politically conservative beliefs, opinions, and values [24].

A number of studies have documented the effects of threat on the endorsement of conservative and system-justifying attitudes [14–18, 25, 26, 27, 28]. At the same time, other studies suggest more complex patterns of interaction between situational and dispositional factors. For example, Banfield and colleagues found that low system justifiers were more likely to defend the system (e.g., supporting reform rather than replacement of the educational system) under high versus low threat [29]. By contrast, high system justifiers were unaffected by the presence or absence of a threat. Cutright and colleagues also observed that only low system justifiers increased their preference for domestic (vs. international) consumer brands following a system-level threat [30]. However, with respect to mortality salience threats, they observed a general shift towards the American system regardless of dispositional levels of system justification, in line with the original hypothesis suggested by Jost and colleagues [19].

### Terror Management Theory

Although a good deal of empirical evidence links the experience of threat to political conservatism [31], some theorists have argued that threat-related shifts in political attitudes are linked to ideological extremity rather than conservatism per se [32]. This argument stems, at least in part, from terror management theory (TMT), which posits that human beings, like all animals, are instinctively driven to engage in self-preservation [33, 34]. However, unlike other living organisms, humans possess an awareness that their own death is inevitable. According to the theory, the combination of survival instinct and mortality awareness creates a potentially debilitating form of anxiety. TMT describes two ways of assuaging this terror: individuals can either bolster their own cultural worldviews (i.e., a socially shared set of beliefs about the nature of reality that prescribes norms of proper conduct and standards of pursuing personal value) or increase their self-esteem (i.e., the perception that one is meeting or exceeding the value standards set forth by one’s cultural worldview). The theory holds that mortality salience primes should increase commitment to one’s own cultural worldview (whether liberal or conservative) as well as the derogation of contrasting worldviews [35–37]. This means that existential threat should produce ideological polarization rather than an overall conservative shift.

The ideological polarization hypothesis has received some empirical support [38–42]. However, nearly all of the studies conducted during the 2004 presidential election documented a general shift in favor of President Bush in response to existential threat [9, 43]. In an effort to explain why relatively liberal college students would become more supportive of Presidential Bush and less supportive of his challenger, John Kerry, TMT researchers began to explore the appeal of charismatic leadership as a qualification of the original TMT prediction of ideological polarization. It was theorized that charismatic leaders allow anxious individuals to feel like valuable parts of a larger whole while providing an inspiring vision of how to overcome threatening circumstances.

In an experimental elaboration, Cohen and colleagues found that mortality salience shifted preferences for hypothetical candidates who displayed charismatic (vs. task-oriented and relationship-oriented) speech styles [20]. Candidates who gave a charismatic (i.e., visionary) speech received seven times more votes in the mortality salience condition than they did in the control condition. Kosloff and colleagues adapted these procedures and also varied the political ideology of the hypothetical candidate [44]. In this experiment, individuals exhibited significantly greater support for charismatic candidates in the mortality salience condition—but only when the candidate “matched” the participant’s political ideology. In the TMT literature this is now referred to as the “charisma-orientation match” hypothesis.

### System Justification vs. Terror Management Theory

There have been previous empirical attempts to pit the predictions of SJT and TMT against one another (e.g., [18, 25, 32, 45, 46]), but these attempts concentrated largely on data collected during the eight years of George W. Bush’s presidency. There are reasons to suspect that this period was an unusual one in terms of U.S. political history because of the September 11^th^ terrorist attacks in 2001, the War in Afghanistan that same year, the War in Iraq in 2003, Hurricane Katrina in 2005, and the financial crisis that began in 2008. This historical concern was expressly voiced by Burke and colleagues [45]:

[I]t appears that historical context can determine whether liberal or conservative values facilitate terror management. When prevailing cultural trends favor conservatism, MS often strengthens conservative leanings; yet when prevailing trends are more progressive in nature, MS often strengthens liberal leanings (p. 11).

We hope to advance the scientific understanding of how social-contextual factors might moderate the effect of existential threat on ideological preferences by capitalizing on a different electoral context, namely the 2012 U.S. Presidential campaign between Democrat incumbent Barack Obama and Republican challenger Mitt Romney. By integrating raw data from four different experimental interventions, we assembled a combined data set that provided a sample that was robust enough to assess multiple theoretical possibilities derived from both SJT and TMT.

We considered the political context of the 2012 election to be especially interesting and theoretically probative for a number of reasons. First, to address concerns raised by Burke and colleagues [45], we sought to compare the competing predictions of SJT and TMT in a context in which the political climate was (at least arguably) less favorable to conservative interests than the previous two or three elections. Given four years of a relatively liberal presidency prior to the 2012 campaign, it was somewhat unclear, from a system justification perspective, which system (or status quo) would be the one that was defended under threat: (1) the “traditional” American system, which supports conservative ideology and resists changes to the status quo, or (2) the incumbent administration, which promoted liberal changes associated with more universal healthcare coverage, legalization of same-sex marriage, and a smaller Defense budget. Second, we were able to investigate more directly the role of charisma in response to mortality salience inductions. President Obama (the more liberal candidate) was clearly perceived to be more charismatic than Romney (see empirical evidence below), and therefore general shifts in either direction would help to distinguish between conservative ideology and charismatic leadership as competing explanations.

Before turning to the different predictions of SJT and TMT, we first note their similarities. Both theories seek to understand the relationship between political ideology and motivational underpinnings; both stress the social construction of ideological realities and the need for consensual validation of ideological beliefs; and both regard social stereotypes as ideological justifications [25]. Furthermore, in many situations, the predictions of both theories coincide. For instance, both theories predict that conservatives should become more conservative in response to threat, and that when nationalistic concerns are salient, individuals might shift towards values that are considered patriotic if not nationalistic.

The points on which the theories differ served as research questions to be examined with the data at our disposal. Specifically, we focused on four diverging and, in some cases, conflicting predictions:

1. *Do liberals become more liberal in response to threat?* SJT predicts that, in response to a mortality salience manipulation, liberals—like conservatives—will generally become more favorable toward the conservative candidate (H1a). By contrast, TMT predicts that liberals will become more favorable toward the liberal candidate (H1b).
2. *Does system justification moderate the effect of threat?* SJT predicts that in the absence of threat, high system justifiers will endorse the conservative candidate more than low system justifiers. Under mortality salience, SJT predicts that low system justifiers will become more favorable toward the conservative candidate. TMT does not offer a prediction when it comes to dispositional system justification.
3. *Are perceptions of charisma more predictive of candidate support in response to threat?* TMT predicts that mortality salience will lead people (regardless of ideology) to exhibit stronger support for a candidate to the extent that they perceive him (or her) to be more charismatic. SJT does not offer a prediction with regard to charisma per se.
4. *Does ideological similarity moderate the effect of charisma in response to threat?* TMT predicts that under mortality salience, liberals will show increased support for Obama (but not Romney) to the extent that they perceive him as more charismatic, whereas conservative participants will show increased support for Romney (but not Obama) to the extent that they perceive him as more charismatic.

Because none of these predictions received unambiguous support, we also conducted a series of exploratory analyses in which we investigated, among other things, possible interaction effects between political ideology and system justification.

### The Present Research

We conducted four roughly concurrent studies one month prior to the 2012 U.S. presidential election. The procedures for all four were very similar. After administering a few introductory scales to obscure the studies’ main focus, we exposed half of the participants to a reminder of their own mortality. Following the manipulation of mortality salience (vs. a control condition), all participants completed some form of a delay task (see Table 1), rated both presidential candidates on a continuous scale of support, and predicted whom they would vote for in the upcoming election. Experimental manipulations were adapted from studies conducted by terror management researchers during the 2004 presidential election cycle [9, 20, 43]. For all analyses, we pooled data from all four samples to maximize statistical power and obtain estimates that would be robust to variations in sampling and experimental procedures.

**Table 1:** Experimental Procedures by Sample

*Sample 1*	*Sample 2*	*Sample 3*	*Sample 4*
MTurk	College classroom	MTurk	MTurk
*N* = 98	*N* = 73	*N* = 211	*N* = 241
Experimental manipulation: mortality salience vs.			
Intense pain	Favorite TV program	Intense pain	Favorite TV program
Delay literary passage			
PANAS			
↓	1 opinion article	1 opinion article	2 speech segments
Support ratings			
Charisma ratings			
Political ideology question			
System justification scale			

## Method

### Participants

#### Sample 1

Ninety-two participants were recruited online using Amazon’s Mechanical Turk (see [47, 48] for descriptions and assessments of this research platform as applied to research in psychology and political science, respectively). One participant was dropped for living in the U.S. for less than two years. The final sample consisted of 91 participants (40 females, 51 males, *M*_*age*_ = 33.35, *SD* = 11.65). Participants were compensated 50 cents for half an hour of participation in the study.

#### Sample 2

Seventy-three participants were recruited in psychology classes at a small liberal arts college in Pennsylvania. Three were dropped for not completing the experimental manipulation. The final sample consisted of 70 participants (45 females, 25 males, *M*_*age*_ = 19.49, *SD* = 1.44). Students participated on a voluntary basis as a learning experience.

#### Sample 3

Two hundred and five participants were recruited through Mechanical Turk. Six were dropped for not completing the experimental manipulation. Twenty-four were dropped for living in the U.S. for less than two years. The final sample consisted of 175 participants (106 females, 68 males, 1 other, *M*_*age*_ = 31.81, *SD* = 10.49). Participants were compensated 50 cents for half an hour of participation in the study.

#### Sample 4

Two hundred and thirty-one participants were recruited through Mechanical Turk. Two were dropped for not completing the experimental manipulation. One was dropped for living in the U.S. for less than two years. The final sample consisted of 228 participants (148 females, 79 males, 1 other, *M*_*age*_ = 36.13, *SD* = 13.81). Participants were compensated 50 cents for half an hour of participation in the study.

### Procedure

We posted an advertisement on Amazon’s Mechanical Turk for an experiment concerning personality and social issues. Surveys were conducted using Qualtrics’ survey platform. Interested participants clicked a link and began the experiment after providing written informed consent. Participants were randomly assigned to either the mortality salience or a control condition. The mortality salience manipulation followed two simple personality questionnaires used in prior research to support the cover story (e.g., [9, 43]). The mortality salience manipulation (n = 285) required participants to respond to two open-ended questions used in prior research: “Please briefly describe the emotions that the thought of your own death arouses in you” and “Jot down, as specifically as you can, what you think will happen to you when you are physically dead.” Participants assigned to the *control condition* (n = 271) responded to parallel questions about being in “Intense Pain” or watching their “Favorite TV Show.” Control conditions were taken from the original 2004 TMT studies on which we modeled our experiments.

Next, all participants completed a self-reported mood scale (PANAS) [49] to assess whether the manipulation evoked negative affect. Because previous research has shown that mortality salience effects are most consistent after a delay [50], participants were asked to read and answer questions concerning a short literary passage before completing a 5-item scale of candidate support for both Obama and Romney and questions about each of the candidates’ levels of charisma. Slight procedural variations (based on the designs of the original 2004 studies) took place either before or after the candidate support ratings (see Table 1). As another measure of candidate support, participants were asked who they thought they would vote for in the upcoming election. Finally, they provided demographic information, reported their political ideology, and completed the General System Justification Scale [51]. All participants were debriefed and thanked for their participation. This research was approved by New York University's Committee on Activities Involving Human Subjects (approval #12–9166).

### Materials

#### Political ideology

Participants’ ideology was measured with one item: “Where on the following scale of political orientation would you place yourself?” (as measured in prior research, e.g., [52]). Responses were provided on a nine-point scale (1 = *Extremely Liberal* to 9 = *Extremely Conservative*).

In the studies presented in this paper, participants were slightly more liberal than conservative in terms of ideology (*M* = 4.37, *SD* = 2.28), but the average was fairly close to the mid-point of 5 (for individual sample descriptives, see Table 2).

**Table 2:** Means, Standard Deviations, and Correlations for Variables by Sample.

Variable	*M*	*SD*	1	2	3
*Sample 1 (N = 98)*					
1. Political Ideology	4.02				
2. System Justification	4.81	1.40	.24*		
3. Perceived Charisma	1.32	1.63	-.53**	-.20	
4. Candidate Support	1.05	2.14	-.78**	-.32**	.71**
*Sample 2 (N = 73)*					
1. Political Ideology	5.51	1.95			
2. System Justification	4.56	1.30	.22		
3. Perceived Charisma	1.22	1.69	-.14	.01	
4. Candidate Support	1.27	1.83	-.46**	-.17	.65**
*Sample 3 (N = 211)*					
1. Political Ideology	4.22	2.16			
2. System Justification	4.78	1.29	.04		
3. Perceived Charisma	1.15	1.69	-.58**	-.09	
4. Candidate Support	1.02	1.98	-.76**	-.04	.74**
*Sample 4 (N = 241)*					
1. Political Ideology	4.10	2.23			
2. System Justification	4.70	1.45	.24**		
3. Perceived Charisma	1.17	1.77	-.49**	-.22**	
4. Candidate Support	1.09	2.17	-.70**	-.30**	.68**

Political Ideology was measured with a single item using a nine-point scale, with higher numbers indicating greater conservatism. System justification was measured with eight items using a nine-point scale, with higher numbers indicating stronger system justifying attitudes. Perceived charisma was measured with a difference score: Obama’s perceived charisma minus Romney’s perceived charisma (both measured with one item on a five-point scale with higher numbers indicating greater perceived charisma). Positive difference scores therefore indicate perceiving higher levels of charisma for Obama than Romney. Candidate support was measured with a difference score of Obama’s candidate support minus Romney’s candidate support (both measured with five items on a five-point scale with higher numbers indicating greater candidate support). Positive difference scores indicate stronger support for Obama than Romney.

^a †^*p* < .10.

* *p* < .05.

** *p* < .01.

Due to a programming error, there was a response option provided that fell outside of the 1–9 range. Eight participants chose this option, which was next to the *Extremely Conservative* response option. To try to determine which, if any, of these eight participants intended to identify themselves as “extremely conservative” (as opposed to treating the 10^th^ response option as a “not applicable” category), we used multiple regression to impute the values of the ambiguous responses. The three cases for which the imputed value was 7 or greater were recoded as 9, assuming that those participants, given their other data, intended to rate themselves as “extremely conservative.” The five other cases were left as missing.

#### Charisma ratings

Each candidate’s perceived charisma was measured with one item: “To what extent do you believe that [Barack Obama/Mitt Romney] is charismatic.” Responses were provided on a 5-point scale (from *Not at all* to *A great deal*). In every sample, participants perceived Obama to be significantly more charismatic than Romney (all *p*s < .001).

#### System justification

Participants completed the 8-item general system justification scale [51]. Participants rated their agreement with statements either supporting or criticizing the system on a 1 to 9 scale from *strongly agree* to *strongly disagree* (α = .80). In the studies presented here, ideology and system justification were positively but modestly correlated (ranging from .04 [n.s.] to .24, *p* < .01) such that individuals who rated themselves as more conservative also scored higher on the system justification scale.

To check whether any of our independent variables were affected by the experimental manipulations, we regressed each independent variable that followed the experimental manipulations on an effect code for mortality salience while adjusting for sample-by-sample variation. Threat did not influence the ideology self-placement, *b* = .05, *SE* = .10, *t*(554) = .49, *p* = .62; charisma ratings, *b* = .01, *SE* = .08, *t*(558) = .15, *p* = .88; or system justification scores, *b* = -.03, *SE* = .07, *t*(558) = -.45, *p* = .66.

#### Candidate ratings

Candidate support was measured with a five-item scale (e.g., “How much do you believe [Barack Obama/Mitt Romney] can contribute to society?”, “How much do you find [Barack Obama/Mitt Romney]’s beliefs in agreement with your own?”, “To what extent would [Barack Obama/Mitt Romney] be an ideal president?”). Responses were provided on a 5-point scale (from *Not at all* to *A great deal*). The internal consistencies of the two scales were excellent (Obama ratings α = .96; Romney ratings α = .95).

#### Voting intentions

Participants also responded to a one-item question to assess voting intentions: “To the best of your knowledge, who do you expect to vote for in the upcoming election?” Participants either chose Barack Obama, Mitt Romney, *other*, or *not sure*.

### Statistical Analysis

We followed the Integrative Data Analysis (IDA) approach of Curran and Hussong [53], and combined the data from the four samples into a single dataset. This approach has several important advantages over piecemeal analyses: (a) it provides more power and precision for investigating the effects of the mortality salience manipulation, in comparison with individual study analyses, (b) it allows a more rigorous control of type I error by reducing the number of tests pertaining to the manipulation, (c) it facilitates a formal analysis of the generalizability of findings across samples and minor procedural variations. These are advantages that are offered by secondary meta-analysis, but IDA allows even more rigor than meta-analysis because the same statistical adjustments and analytic methods can be applied to different samples. In addition to the central IDA results, we report study-specific results for archival purposes.

Specifically, we implemented the IDA by building general linear models that predicted candidate preference as a function of mortality salience manipulation, ideology, system justification, and their interactions across the four samples. In addition we treated the samples as fixed effects, which means that we incorporated sample membership as a known and fixed characteristic of each participant. The coding of sample source is then used in subsequent analyses in a manner that is similar to the treatment of participant sex or experimental condition. One advantage to this strategy is that all between-sample variability can be eliminated, even when specific measures that describe these differences are not available.

In addition to investigating hypothesized relations among mortality salience, ideology, and system justification, we also examined interactions of these effects by sample source. We did not have hypotheses about the nature of possible differences, and we noted that the number of these exploratory tests are large, making it likely that we would find a difference by chance alone. For this reason we implemented a Benjamini-Hochberg adjustment, which controls for the false discovery rate, that is, the expected percentage of false predictions in a given set of predictions, on all unpredicted *p*-values [54, 55]. The B-H procedure controls for false discovery rate by sequentially comparing rank-ordered observed *p*-values from a family of tests. This procedure is conceptually similar to the Bonferroni correction, but it yields greater power in its computation. All exploratory results that we report were significant even after the B-H adjustment.

## Results

### Candidate Ratings

As expected, the support ratings for Obama and Romney were negatively correlated, *r*s ranged from -.49 to -.63, *p*s < .01. For both candidates, perceived charisma and support ratings were strongly and positively correlated: for Obama, *r*s ranged from .52 to .69; for Romney, *r*s ranged from .50 to .66, *p*s < .01. These correlations indicate that while there is a strong relationship between support and the perception of charisma, they are not identical constructs. To address the first two research questions, a difference score was created by subtracting Romney’s support ratings from Obama’s to obtain a single comparative measure of candidate support (α = .98), with zero indicating equal support. In all four studies there was more support for Obama than Romney; the average support over the four samples was 1.08 (*95% CI* [.91, 1.25]). To address the third and fourth research questions, we modeled the candidate ratings in repeated measures format, including which candidate was being evaluated as a predictor in the model. The rationale for the expanded model will be discussed below.

### Testing Diverging Predictions of TMT and SJT

Table 3 reports a series of tests of the multivariate associations involving mortality salience, system justification, and political ideology in relation to the contrast of support for Obama versus Romney. These tests provide information that is relevant to the different predictions of TMT and SJT with regard to mortality salience and relative support for these two candidates. The tests are based on Type III sums of squares, which essentially gives equal weight to the four individual studies.

**Table 3:** Model Predicting Relative Presidential Support for Obama vs. Romney.

*Predictor*	*df*	*SS*	*MS*	*F*	*p*
Intercept	1	518.21	518.21	260.01	0.00
System Justification	1	0.21	0.21	0.11	0.75
Political Ideology	1	633.47	633.47	317.83	0.00
System Justification * Ideology	1	40.27	40.27	20.21	0.00
Sample	3	35.33	11.78	5.91	0.00
System Justification * Sample	3	21.18	7.06	3.54	0.02
Ideology * Sample	3	5.59	1.87	0.94	0.42
System Justification * Ideology * Sample	3	21.76	7.25	3.64	0.01
Mortality Salience	1	1.76	1.76	0.88	0.35
Mortality Salience * System Justification	1	0.32	0.32	0.16	0.69
Mortality Salience * Ideology	1	2.64	2.64	1.33	0.25
Mortality Salience * System Justification * Ideology	1	2.57	2.57	1.29	0.26
Mortality Salience * Sample	3	2.64	0.88	0.44	0.72
Mortality Salience * System Justification * Sample	3	23.21	7.74	3.88	0.01
Mortality Salience * Ideology * Sample	3	11.73	3.91	1.96	0.12
Mortality Salience * System Justification * Ideology * Sample	3	4.47	1.49	0.75	0.52
Error	1040.38	522	1.99		
Total	2341.70	553			

Political Ideology was measured with one item on a nine-point scale where higher numbers indicate greater conservatism. System justification was measured with eight items on a nine-point scale where higher numbers indicate stronger system justifying attitudes. Candidate support was measured with a difference score of Obama’s candidate support minus Romney’s candidate support (both measured with five items on a five-point scale where higher numbers indicate greater candidate support). Higher numbers on the difference score indicate exhibiting greater support towards Obama than towards Romney.

#### Question 1: *Do liberals become more liberal in response to threat?*

TMT predicts that mortality salience threat will make liberals more likely to support the liberal candidate, whereas SJT predicts the opposite—that liberals as well as conservatives will become more supportive of the conservative candidate following mortality salience threat. This means that SJT would predict a main effect of mortality salience that shifts all participants towards the more conservative candidate—Romney in the case of the 2012 election. When results were combined across all four studies, we did not observe any statistically significant evidence of a mortality salience effect on candidate support ratings, *F*(1, 522) = .88, *p* = .35 (see Table 3). Support for Obama was .15 points non-significantly lower in the mortality salience condition (1.20) than in the control condition (1.35; see Appendix A in S1 Text for the regression model with coefficients and confidence intervals).

The two-way interaction between mortality salience and political ideology was also non-significant, *F*(1, 522) = 1.33, *p* = .25, contrary to the TM prediction. For participants who were one standard deviation more liberal than the average, the preference for Obama over Romney was only slightly larger in the control condition (2.84) than in the mortality salience condition (2.51), and this difference is consistent with sampling variation. For conservatives, the preference for Romney over Obama was slightly greater in the control condition (-.14) than in the mortality salience condition (-.10), but not significantly greater. In other words, being in the mortality salience condition weakened the relationship between political ideology and candidate support by .08 as compared to being in the control condition.

Therefore regarding the first set of predictions, we did not find support for either SJT or TMT. There was no consistent shift in favor of Romney following mortality salience, and the effect of mortality salience threat was not consistently different for liberals versus conservatives (see Appendix B in S1 Text for confidence interval and effect size estimates).

#### Question 2: *Does system justification moderate the effect of threat?*

SJT suggests that the effect of mortality salience may be more pronounced for persons who are chronically low (vs. high) in system justification. More specifically, SJT predicts movement toward the candidate who most represents the system, and we generally interpret this as movement toward the more conservative candidate, in this case Romney. To test this prediction, we evaluated the interaction of morality salience and system justification. The interaction was not significant, *F*(1, 522) = .16, *p* = .69. For people who scored one standard deviation below the mean on system justification, preference for Obama over Romney was only slightly greater in the control condition (1.41) than in the mortality salience condition (1.19). This pattern held for the people who scored one standard deviation above the mean on system justification: preference for Obama over Romney was non-significantly greater in the control condition (1.29) than in the mortality salience condition (1.21). Being in the mortality salience condition diminished the relationship between system justification and candidate support by .05 in comparison with being in the control condition, but this effect is consistent with sampling fluctuation.

**Fig 1:**
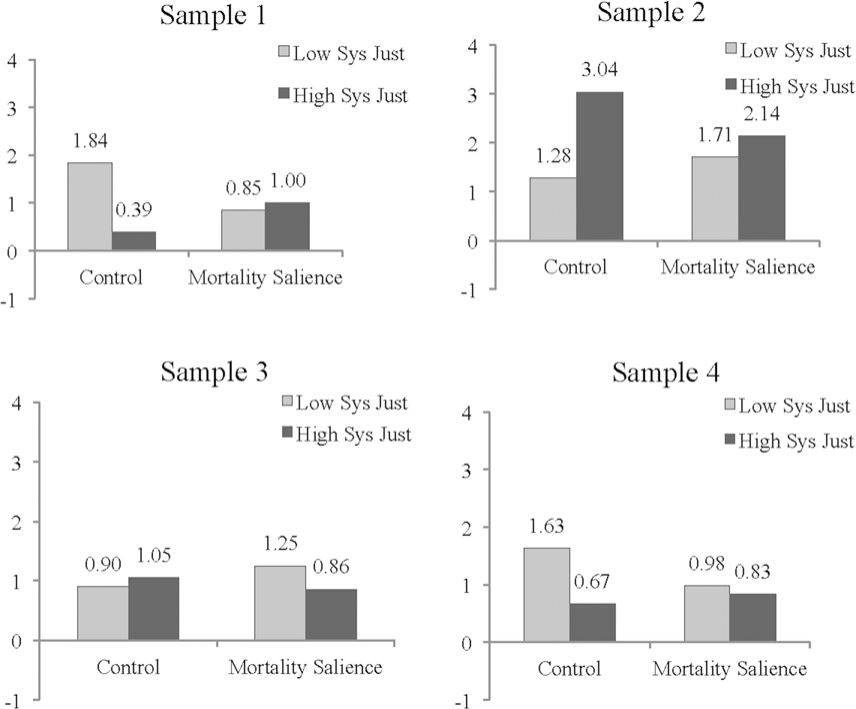
Interaction of Sample, Mortality Salience, and System Justification Predicting Candidate Support (Obama ratings–Romney ratings). “Low System Justifiers” represents one standard deviation below the mean of system justification; “High System Justifiers” represents one standard deviation above the mean of system justification.

Table 3 reveals an important qualification to this conclusion. The strength of the interaction of mortality salience with system justification varied significantly over the four samples *F*(3, 522) = 3.88, *p* = .009. To better understand the sample-by-sample variation, we reanalyzed the data to assess the interaction of mortality salience by system justification for each individual sample (see Appendix C in S1 Text for full tables of sample-level results). To ease interpretation, we standardized each continuous variable within sample so that one unit referred to a standard deviation unit. There were statistically significant interactions in samples 1 and 4, *b* = .19, *SE* = .08, *t*(522) = 2.49, *p* = .01 and *b* = .10, *SE* = .05, *t*(522) = 2.08, *p* = .04, respectively, and these both followed the predicted direction (see Fig 1). For both samples, low system justifiers showed stronger support for Obama (over Romney) than high system justifiers in the control condition, *b* = -.35, *SE* = .10, *t*(522) = -3.31, *p* = .001 and *b* = -.23, *SE* = .07, *t*(522) = -3.40, *p* = .001. Under morality salience, however, low and high system justifiers did not differ from one another in terms of candidate preferences, *p*s > .5. This is because mortality salience caused low system justifiers to be less supportive of Obama, *b* = -.22, *SE* = .11, *t*(522) = -2.01, *p* = .045 and *b* = -.16, *SE* = .07, *t*(522) = -2.29, *p* = .02. High system justifiers were unaffected by the mortality salience manipulation. In samples 2 and 3, the interaction of mortality salience and system justification did not approach significance, *p*s > .15. Although the combined evidence did not support predictions made on behalf of SJT, the fact that the predicted pattern appeared in two of the four studies is noteworthy.

#### Question 3: *Are perceptions of charisma more predictive of candidate support in response to threat?*

According to TMT, people who are exposed to mortality salience will exhibit stronger support for candidates who are perceived to be more charismatic, especially when the candidate shares their ideological orientation. To investigate this prediction, we carried out an analysis in which the dependent variable was support for an individual candidate (rather than the difference score DV in Table 3) and perceived charisma for that candidate was one of the independent variables. Because respondents rated both Romney and Obama, we included both ratings as repeated measures in an expanded model using a mixed model approach (see Table 4). Restructuring our analysis in this way allowed us to associate each candidate’s perceived charisma with his support ratings while maintaining a unified model for both candidates. The interactions of candidate with other variables, such as system justification and mortality salience, provide tests that are comparable to the difference score approach in Table 3, but in this case the results are adjusted for the charisma ratings of the individual candidates. We examined the dependency of the two support ratings and concluded that a mixed model that allowed the residuals to be correlated was a better approach [56] than a model specifying subject as a random effect. The residuals of Obama’s and Romney’s support ratings were negatively correlated. All of the results reported were adjusted for this correlation. The drawback to the repeated measures structure as compared to the difference score dependent measure model in Table 3 is that it adds another parameter at every step of the model and thus requires more care in the interpretation of model results.

**Table 4:** Predicting Presidential Support with an Expanded Model.

Predictor	Num df	Den df	F	p
Intercept	1	626.46	5313.59	0.00
Candidate	1	745.33	88.48	0.00
System Justification * Candidate	1	706.53	0.27	0.60
Ideology * Candidate	1	721.72	186.60	0.00
Ideology * System Justification * Candidate	1	735.10	11.84	0.00
Sample * Candidate	3	732.46	4.66	0.00
Sample * System Justification * Candidate	3	712.45	1.91	0.13
Sample * Ideology * Candidate	3	689.16	0.07	0.98
Sample * Ideology * System Justification * Candidate	3	723.26	3.66	0.01
Mortality Salience * Candidate	1	745.33	1.55	0.21
Mortality Salience * System Justification * Candidate	1	706.53	1.07	0.30
Mortality Salience * Ideology * Candidate	1	721.72	2.31	0.13
Mortality Salience * Perceived Charisma * Candidate	1	703.23	0.66	0.42
Mortality Salience * Ideology * Perceived Charisma * Candidate	1	751.57	1.47	0.23
Sample * Mortality Salience * Candidate	3	732.46	1.09	0.35
Sample * Mortality Salience * System Justification * Candidate	3	712.45	1.35	0.26
Sample * Mortality Salience * Ideology * Candidate	3	689.16	1.25	0.29
Sample * Mortality Salience * Perceived Charisma * Candidate	3	746.81	0.80	0.49
Sample * Mortality Salience * Ideology * Perceived Charisma * Candidate	3	761.43	0.44	0.72
Mortality Salience	1	626.46	0.74	0.39
Ideology	1	635.94	0.52	0.47
Perceived Charisma	1	770.83	97.51	0.00
System Justification	1	626.14	31.75	0.00
Mortality Salience * Ideology	1	635.94	0.01	0.94
Mortality Salience * Perceived Charisma	1	770.83	1.41	0.24
Mortality Salience * System Justification	1	626.14	0.01	0.95
Ideology * Perceived Charisma	1	867.30	1.84	0.18
Ideology * System Justification	1	654.20	0.24	0.62
Perceived Charisma * System Justification	1	722.47	3.01	0.08
Perceived Charisma * Candidate	1	703.23	6.01	0.01
Mortality Salience * Ideology * Perceived Charisma	1	867.30	0.38	0.54
Mortality Salience * Ideology * System Justification	1	654.20	0.15	0.70
Mortality Salience * Perceived Charisma * System Justification	1	722.47	2.37	0.12
Ideology * Perceived Charisma * System Justification	1	859.42	0.88	0.35
Ideology * Perceived Charisma * Candidate	1	751.57	15.42	0.00
Perceived Charisma * System Justification * Candidate	1	755.40	2.44	0.12
Mortality Salience * Ideology * Perceived Charisma * System Justification	1	859.42	0.07	0.79
Mortality Salience * Ideology * System Justification * Candidate	1	735.10	0.13	0.72
Mortality Salience * Perceived Charisma * System Justification * Candidate	1	755.40	0.08	0.78
Ideology * Perceived Charisma * System Justification * Candidate	1	783.70	0.03	0.86
Mortality Salience * Ideology * Perceived Charisma * System Justification * Candidate	1	783.70	0.32	0.57
Sample	3	617.53	1.23	0.30
Sample * Mortality Salience	3	617.53	0.63	0.59
Sample * Ideology	3	604.70	1.27	0.28
Sample * Perceived Charisma	3	771.64	0.81	0.49
Sample * System Justification	3	603.60	1.80	0.15
Sample * Mortality Salience * Ideology	3	604.70	0.93	0.43
Sample * Mortality Salience * Perceived Charisma	3	771.64	1.18	0.32
Sample * Mortality Salience * System Justification	3	603.60	1.20	0.31
Sample * Ideology * Perceived Charisma	3	876.90	1.15	0.33
Sample * Ideology * System Justification	3	627.90	1.65	0.18
Sample * Perceived Charisma * System Justification	3	741.11	0.51	0.67
Sample * Perceived Charisma * Candidate	3	746.81	0.86	0.46
Sample * Mortality Salience * Ideology * Perceived Charisma	3	876.90	0.72	0.54
Sample * Mortality Salience * Ideology * System Justification	3	627.90	0.23	0.87
Sample * Mortality Salience * Perceived Charisma * System Justification	3	741.11	0.48	0.69
Sample * Ideology * Perceived Charisma * System Justification	3	850.22	1.27	0.28
Sample * Ideology * Perceived Charisma * Candidate	3	761.43	0.60	0.61
Sample * Perceived Charisma * System Justification * Candidate	3	759.07	1.63	0.18
Sample * Mortality Salience * Ideology * Perceived Charisma * System Justification	3	850.22	0.16	0.92
Sample * Mortality Salience * Ideology * System Justification * Candidate	3	723.26	0.57	0.63
Sample * Mortality Salience * Perceived Charisma * System Justification * Candidate	3	759.07	2.08	0.10
Sample * Ideology * Perceived Charisma * System Justification * Candidate	3	728.47	1.06	0.37

Political Ideology was measured with one item on a nine-point scale where higher numbers indicate greater conservatism. System justification was measured with eight items on a nine-point scale where higher numbers indicate stronger system justifying attitudes. Perceived Charisma was measured with one item on a five-point scale where higher numbers indicate greater perceived charisma. Candidate support was measured with five items on a five-point scale where higher numbers indicate greater candidate support.

In all samples, Obama was perceived to be more charismatic than Romney, *p*s < .001. Both conservatives, identified at one standard deviation above the mean of political ideology (Obama’s *M* = 3.70, Romney’s *M* = 3.31), and liberals, identified at one standard deviation below the mean of political ideology (Obama’s *M* = 4.54, Romney’s *M* = 2.53), perceived Obama to be more charismatic than Romney. Charisma was related to support for either candidate (*F*(1, 788.9) = 125.44, *p* < .001), with support increasing by *b* = 0.41 (*SE* = .037) units for each unit change in perceived charisma. However, the hypothesized interaction between mortality salience and perceived charisma was not significant, *F*(1, 807.7) = .97, *p* = .33. There was also no evidence of the mortality salience by charisma effect interacting with candidate, *F*(1,769.1) = .65, *p* = .42.

#### Question 4: *Does matching moderate the effect of charisma in response to threat?*

Given that Obama was perceived to be the more charismatic candidate, the orientation-match hypothesis suggests that only liberals would exhibit increased support for Obama under mortality salience. Conservatives, on the other hand, should exhibit increased support for Romney under mortality salience—but only to the degree that they perceived Romney to be more charismatic than Obama. Perhaps individuals who perceived the candidate who did not share their political ideology to be more charismatic would be even less supportive under mortality salience. To address these possibilities, we utilized the same expanded model reported above and checked for a four-way interaction involving mortality salience, perceived charisma, political ideology, and candidate. This interaction did not approach statistical significance, *F*(1, 751.57) = 1.47, *p* = .23.

### Exploratory analyses

Given the lack of support for most of the predictions put forth by terror management and system justification theories, we examined associations in the data that were not part of our formal predictions to better understand the failure to “replicate” findings from previous election cycles. As expected, the main effect of ideology was strong and reliable across all studies and indicated that conservatives were much more likely to support Romney and liberals were more likely to support Obama, *F*(1, 522) = 317.83, *p* < .001.

**Fig 2:**
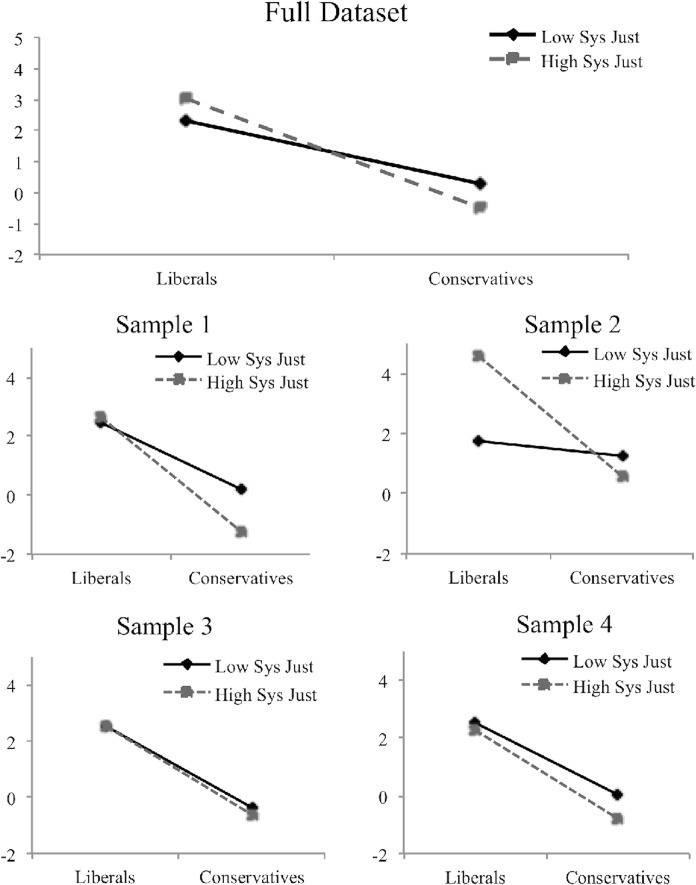
Interaction of Sample, Political Ideology, and System Justification Predicting Candidate Support (Obama ratings–Romney ratings). “Low System Justifiers” represents one standard deviation below the mean of system justification; “High System Justifiers” represents one standard deviation above the mean of system justification. “Liberals” represents one standard deviation below the mean of political ideology; “Conservatives” represents one standard deviation above the mean of political ideology.

We also observed an intriguing but unpredicted two-way interaction between system justification and political ideology, *F*(1, 522) = 20.21, *p* < .001. This two-way interaction was modified by a three-way interaction involving sample, *F*(3, 522) = 3.64, *p* = .01 (see Fig 2). In interpreting this interaction, we begin by analyzing the overall pattern in the data evidenced by the two-way interaction and then proceed to decomposing the sample-by-sample variation. Across all samples, we observed that for conservatives, higher system justification levels significantly predicted stronger support for Romney over Obama, *b* = -.29, *SE* = .07, *t*(522) = -4.21, *p* < .001. For liberals, however, we find the opposite trend, so that higher system justification levels significantly predicted stronger support for Obama over Romney, *b* = .25, *SE* = .10, *t*(522) = 2.48, *p* = .01. For both high and low system justifiers, ideology continued to predict candidate support such that liberals were more likely to support Obama and conservatives were more likely to support Romney, *b* = -.80, *SE* = .05, *t*(522) = -15.17, *p* < .001, and *b* = -.47, *SE* = .05, *t*(522) = -9.21, *p* < .001, respectively.

Focusing on the variation by sample in Fig 2, in Sample 2 (the student sample) we observed trends that differed from the other three samples. In Sample 2, system justification significantly predicted support for Obama among liberals (*b* = 1.05, *SE* = .35, *t*(522) = 3.03, *p* = .003), but it did not significantly predict support for Romney among conservatives, *p* > .1. In all other plots, we observed a strong main effect of political ideology, with generally minor moderation by system justification. This moderation was significant in Sample 1, *b* = -.13, *SE* = .05, *t*(522) = -2.65, *p* = .01, but with a pattern that was different from Sample 2. The pattern in Sample 1 indicates that system justification scores were unrelated to liberals’ candidate ratings, *p* = .73, but system justification scores strongly predicted candidate ratings for conservatives, *b* = -.52, *SE* = .15, *t*(522) = -3.28, *p* = .001, such that conservatives who were high in system justification exhibited a stronger preference for Romney over Obama in comparison with conservatives who were low in system justification. For samples 3 and 4, there was no pronounced version of either pattern, but when the four studies were averaged together the tendency for system justification to amplify support for one’s own ideological preferences emerged. The type of control condition (intense pain vs. favorite TV program) was unrelated to sampling variation.

One explanation for the differences exhibited by Sample 2 may have to do with demographic characteristics. Whereas the other three samples were recruited through Amazon’s Mechanical Turk, Sample 2 was drawn from a small, rural liberal arts college and was largely comprised of college freshmen. These participants were younger and more similar to one another than the participants in the MTurk samples. In addition, they completed the materials in a controlled classroom setting rather than over the internet. Other potentially relevant particularities of this sample are that the participants exhibited the lowest trends in system justification, as well as the highest levels of self-reported conservatism and—at the same time—support for President Obama (as opposed to Governor Romney); this was a unique combination, to say the least.

### Voting intentions

A 2 x 4 chi-square test was computed with mortality salience condition and voting intentions. In contrast to prior findings [43], candidate selection did not differ based on experimental condition, *χ*^*2*^(3, *N* = 560) = 0.26, *p* = .61. The relationship did not vary when accounting for sample-by-sample variation.

### Comparing Effect Sizes with Previous Research

The 2004 studies that inspired us reported effect sizes ranging from relatively small to extremely large effect sizes, *d* = .23–1.50, for the main effect of mortality salience on political preferences. In our integrative data analysis, we estimated a nonsignificant effect size of *d* = .03. Our effect size is consistent with an effect in the population as large as .14 and as small as -.20 (computed with a confidence interval of 95%).

Even the smallest effect size obtained in previous research is outside of the confidence bounds of the effect we observed in the current set of analyses. Although we cannot be certain about what may be responsible for the discrepancy in the effect of morality salience on candidate support, we note a variety of contextual differences between the 2004 election and the one that took place in 2012: a greater temporal distance from the events of September 11, 2001 might have reduced the psychological impact of the mortality salience manipulation; a liberal, biracial incumbent may not “represent” the system or inspire national pride or support in the face of existential threat to the same degree as a conservative, white incumbent; or there may have been something unique about the personality or presidency of George W. Bush (e.g., his pro-war stances, his southern cowboy image, etc.) that made him appealing—even to liberals—in circumstances of high threat.

## General Discussion

In an integrated analysis, we examined four diverging predictions derived from the literatures on terror management theory and system justification theory. Our initial intention was (1) to answer lingering questions about the role of political ideology, perceived charisma, and system justification in response to mortality salience threats, and (2) to establish which hypotheses from each theory would predict outcomes observed during the 2012 election cycle. We expected to conceptually replicate at least some of the previous findings, but the dearth of confirmed predictions and considerable variation among samples led us to consider other possibilities about how to analyze the data.

Among the four predictions derived from terror management and system justification theories, we found partial evidence for only one prediction. Specifically, the asymmetrical shift hypothesis predicts that in the absence of threat, high system justifiers would endorse the conservative candidate more than low system justifiers. However, in response to threat, this hypothesis suggests that *only* low system justifiers would increase their degree of support for the conservative candidate (thus resembling high system justifiers). In two of the four samples, we observed the predicted pattern, but the interaction pattern differed in the two other samples.

It is possible that subtle differences between the experimental designs used in the two samples in which the effect is present and the two in which it is absent could be responsible for these differing patterns of response. In Sample 1, participants engaged in the mortality salience manipulation, read an irrelevant literary piece designed to induce a delay, completed the PANAS, and immediately proceeded to the candidate ratings. In Sample 4, participants were given two short speech segments (one segment attributed to each candidate) before they completed their candidate ratings. In Samples 2 and 3, however, participants were given one (strongly supportive) opinion piece about one of the candidates to read before completing candidate ratings (see Table 1). It is possible that receiving an article slanted in favor of one candidate altered the effect found in Samples 1 and 4.

In previous research, mortality salience threats have evoked some of the strongest situational effects on political attitudes and behaviors, in comparison with other threats [19, 45]. In the current research, neither the main effect of mortality salience nor the interaction of mortality salience by political ideology approached significance. Furthermore, the effect size for the main effect of mortality salience was significantly smaller than the effect sizes observed in previous studies with similar procedures and dependent measures. It is possible that neither terror management nor system justification theories are, in their present incarnations, specified precisely enough to make accurate predictions concerning the effects of threat on political attitudes in circumstances that are anomalous by historical standards, such as U.S. presidential campaigns featuring a relatively charismatic, mixed race, liberal-Democratic incumbent. It is unclear, in any case, how to interpret the differences between the results of studies carried out in 2004 and 2008 and those we obtained in 2012. These differences may—or may not—suggest that something fundamental has shifted in the U.S. political context since the presidency of George W. Bush.

One piece of evidence that might help to explain why the current analyses differ from previous research comes from a serendipitous result: the significant interaction between political ideology and system justification. When beginning this project, we struggled with the question of which candidate in 2012 would more clearly represent the American political system or societal status quo. President Obama, the incumbent, would seem to be the obvious choice, but because of his liberal orientation, and perhaps his mixed race and international pedigree, participants might have viewed him as a departure from the traditional American system (see also [57]). Romney, though less experienced in federal government, may have felt more like the continuation of the American system in that he represented the Republican party, which tends to support conservative policies that promote stability and tradition over change.

While the interaction between political ideology and system justification does exhibit sample-by-sample variation, there was some evidence in our data that participants may have disagreed with one another as to which candidate better represented the system. We found that among liberals, high system justifiers supported Obama more enthusiastically than low system justifiers, and among conservatives, high system justifiers supported Romney more enthusiastically than low system justifiers. In other words, high system justifiers may have chosen which candidate better represented “the system” based on their liberal-conservative ideology. It is possible, given this evidence, that the lack of clear mortality salience effects in the present research may be due to disagreement among participants about which candidate better represented the American system.

For our purposes, analyzing a pooled data set provides a number of advantages over considering the individual samples separately [53]. First, by examining whether statistically significant results were reliable across samples, we were able to evaluate the robustness of the hypothesized pattern of results across studies. Second, the incorporation of sample-by-sample heterogeneity into the analysis allowed us to consider meaningful sources of variability that might contribute to the psychological phenomena under investigation. Third, we wanted to test the hypotheses predicted by the logic of system justification and terror management theories in a manner that was not contingent on any particular sample design. Fourth, given that self-identified conservatives made up our smallest ideological group, pooling the data allowed us to make more reliable predictions about this theoretically important group.

Given the current focus on replication and reproducibility in the field of social and political psychology, the results of our analyses might encourage other researchers to incorporate sample variation in their analyses through integrative data analysis or an analogous strategy. In the present research, we found marked variations in predicted effects based on variations in sample demographics as well as slight procedural differences. The analysis of this sample variation will lead to further refinement of our understanding of contextual moderators and effect stability that cannot be sufficiently brought to light without analyses that incorporate findings across samples.

Taken as a whole, these results should stimulate future research to explore how mortality salience and system justification motivation might work together to influence political attitudes and behavior. In doing so, we encourage four areas of further investigation. First, it is possible that system justification effects may vary depending on the level or type of threat (e.g., personal, group, system) administered. Perhaps in the salient post-9/11 environment of the 2004 election, a mortality salience manipulation functioned both as an individual- and a system-level threat (given the specter of terrorism-related attacks). This association was likely to have been substantially weakened by the year 2012. Second, we found that the interaction of mortality salience by system justification was attenuated when additional information about either candidate was presented after the threat manipulation. Further exploration of the extent to which additional political information can moderate how individuals respond to threat is an area of both great theoretical and practical value. Third, some prominent social scientists argue that it is relatively unlikely to shift candidate support in U.S. presidential elections, given the stability and strength of political views in this contest [58]. It is conceivable that studies conducted at either state or local levels might provide greater variance in responses and thus increase the likelihood of observing shifts in candidate support. Fourth, our data suggest that there may be disagreements about which candidate best represents the national system. It would be useful in future research to probe this possibility further by explicitly asking participants to rank order each candidate in terms of the degree to which he or she represents the American system or “way of life.” Whether or not there is consensus about this in any given election might be an important moderating variable when it comes to the effects of system justification motivation.

The 20^th^ century featured several unforgettable examples of how large-scale national and international threats dramatically changed social and political realities. In the 21^st^ century, we can already guess at some of the conflicts and struggles that have the potential to produce to similarly destabilizing outcomes: the increased scale and frequency of natural disasters due to global climate change, the revolutionary vigor presently dominating Middle Eastern politics, the opportunity for large-scale domestic and international terrorist attacks. Pinpointing the social and political implications of individual-, group-, and system-level threats is as important now as it has ever been.

## Supporting Information

S1 Text Appendix A. Model Predicting Presidential Support with Coefficients and Confidence Intervals. Appendix B. Confidence Interval and Effect Size Estimates. Appendix C. Regression Tables by Sample. Appendix D. Expanded model predicting support for candidates as a function of candidate identity (Obama vs. Romney), participant’s system justification and ideology scores, participants rating of candidate charisma, and whether the participant was in the mortality salience condition.
(DOCX)
