# Supplementary material for: Mortality Salience, System Justification, and Candidate Evaluations in the 2012 U.S. Presidential Election
Source: PLoS One. 2016 Mar 16;11(3):e0150556. doi: 10.1371/journal.pone.0150556 (PMC4794238; doi:10.1371/journal.pone.0150556)
Supplement: S1 Text — Appendix A. Model Predicting Presidential Support with Coefficients and Confidence Intervals. Appendix B. Confidence Interval and Effect Size Estimates. Appendix C. Regression Tables by Sample. Appendix D. Expanded model predicting support for candidates as a function of candidate identity (Obama vs. Romney), participant’s system justification and ideology scores, participants rating of candidate charisma, and whether the participant was in the mortality salience condition. (DOCX) [file pone.0150556.s001.docx]

**Appendix A – Model Predicting Presidential Support with Coefficients and Confidence Intervals**

| *Predictor* | *b* | *SE* | *t* | *p* | *95% CI Lower* | *95% CI Upper* |
| --- | --- | --- | --- | --- | --- | --- |
| Intercept | 1.28 | 0.08 | 16.13 | 0.00 | 1.12 | 1.43 |
| Mortality Salience | -0.15 | 0.16 | -0.94 | 0.35 | -0.46 | 0.16 |
| Ideology | -0.63 | 0.04 | -17.83 | 0.00 | -0.70 | -0.56 |
| System Justification | -0.02 | 0.06 | -0.32 | 0.75 | -0.14 | 0.10 |
| Mortality Salience * Ideology | 0.08 | 0.07 | 1.15 | 0.25 | -0.06 | 0.22 |
| Mortality Salience * System Justification | 0.05 | 0.12 | 0.40 | 0.69 | -0.20 | 0.29 |
| System Justification * Ideology | -0.12 | 0.03 | -4.50 | 0.00 | -0.18 | -0.07 |
| Mortality Salience * System Justification * Ideology | 0.06 | 0.06 | 1.14 | 0.26 | -0.05 | 0.17 |
| Sample 1 | -0.26 | 0.14 | -1.88 | 0.06 | -0.53 | 0.01 |
| Sample 2 | 0.76 | 0.18 | 4.19 | 0.00 | 0.41 | 1.12 |
| Sample 3 | -0.26 | 0.11 | -2.37 | 0.02 | -0.48 | -0.05 |
| Sample 1 * Mortality Salience | -0.04 | 0.27 | -0.15 | 0.88 | -0.58 | 0.50 |
| Sample 1 * System Justification | -0.22 | 0.10 | -2.12 | 0.03 | -0.41 | -0.02 |
| Sample 1 * Ideology | -0.06 | 0.06 | -0.98 | 0.33 | -0.19 | 0.06 |
| Sample 2 * Mortality Salience | -0.09 | 0.37 | -0.24 | 0.81 | -0.81 | 0.63 |
| Sample 2 * System Justification | 0.42 | 0.15 | 2.82 | 0.01 | 0.13 | 0.72 |
| Sample 2 * Ideology | 0.12 | 0.08 | 1.45 | 0.15 | -0.04 | 0.27 |
| Sample 3 * Mortality Salience | 0.23 | 0.22 | 1.02 | 0.31 | -0.21 | 0.66 |
| Sample 3 * System Justification | -0.02 | 0.09 | -0.27 | 0.79 | -0.20 | 0.15 |
| Sample 3 * Ideology | -0.06 | 0.05 | -1.07 | 0.28 | -0.15 | 0.05 |
| Sample 1 * Mortality Salience * System Justification | 0.54 | 0.20 | 2.64 | 0.01 | 0.14 | 0.93 |
| Sample 1 * Mortality Salience * Ideology | -0.30 | 0.13 | -2.38 | 0.02 | -0.55 | -0.05 |
| Sample 1 * System Justification * Ideology | -0.01 | 0.04 | -0.17 | 0.86 | -0.10 | 0.08 |
| Sample 2 * Mortality Salience * System Justification | -0.54 | 0.30 | -1.80 | 0.07 | -1.12 | 0.05 |
| Sample 2 * Mortality Salience * Ideology | 0.16 | 0.16 | 1.03 | 0.30 | -0.15 | 0.48 |
| Sample 2 * System Justification * Ideology | -0.17 | 0.07 | -2.60 | 0.01 | -0.30 | -0.04 |
| Sample 3 * Mortality Salience * System Justification | -0.25 | 0.17 | -1.41 | 0.16 | -0.59 | 0.10 |
| Sample 3 * Mortality Salience * Ideology | 0.10 | 0.10 | 1.02 | 0.31 | -0.10 | 0.30 |
| Sample 3 * System Justification * Ideology | 0.10 | 0.04 | 2.71 | 0.01 | 0.03 | 0.18 |
| Sample 1 * Mortality Salience * System Justification * Ideology | 0.09 | 0.09 | 1.05 | 0.30 | -0.08 | 0.27 |
| Sample 2 * Mortality Salience * System Justification * Ideology | -0.03 | 0.13 | -0.20 | 0.85 | -0.29 | 0.23 |
| Sample 3 * Mortality Salience * System Justification * Ideology | -0.08 | 0.08 | -1.10 | 0.27 | -0.23 | 0.07 |

*Note.* Political Ideology was measured with one item on a nine-point scale where higher numbers indicate greater conservatism. System justification was measured with eight items on a nine-point scale where higher numbers indicate stronger system justifying attitudes. Mortality salience was effect coded (-0.5 for the control condition, .5 for the mortality salience condition). Sample was effect coded with Sample 4 serving as the reference group. Candidate support was measured with a difference score of Obama’s candidate support minus Romney’s candidate support (both measured with five items on a five-point scale where higher numbers indicate greater candidate support). Higher numbers on the difference score indicate exhibiting greater support towards Obama than towards Romney.

**Appendix B – Confidence Interval and Effect Size Estimates**

Because of the large number of null results in this article, we computed confidence intervals involving all major hypotheses to delineate the range of effect sizes that are consistent with the data we obtained.

**Main effect of mortality salience.** Confidence bounds on the estimate reveal that the data are consistent with no mortality salience effect, as well as a mortality salience effect as small as -.16 and as large as .46 (see Appendix A for the regression model with coefficients and confidence intervals). Translated into Cohen’s *d* effect size, our data are consistent with a Cohen’s *d* effect size of .22, which tends to be categorized as small, but not with larger effects. Therefore, our data are inconsistent with medium or large effect sizes, but they are not conclusive in regards to small mortality salience effects.

**Interaction of mortality salience by political ideology.** The data are consistent with no mortality salience by ideology interaction, but also consistent with an effect as small as -.06 and as large as .22. In the metric of Cohen’s *d* effect size, the data are consistent with an interaction effect of .11, which can be categorized as very small. In summary, our data do not support small to large effect sizes, but they are inconclusive in regards to very small mortality salience by ideology interaction effects.

**Interaction of mortality salience by system justification.** The data are consistent with a mortality salience by system justification interaction effect of zero and also as small as -.20 and as large as .29. Therefore, our data are inconsistent with small to large effect sizes, but are inconclusive in regards to very small mortality salience by system justification interaction effects.

**Interaction of mortality salience by perceived charisma.** To determine how conclusive is the negative result for mortality salience by charisma, we examined the confidence bound for this interaction. The interaction point estimate was .072, but the 95% confidence bound ranged from -.07 to .22. The upper bound is of special interest, since it is in the predicted direction. The data are consistent with mortality salience strengthening the effect of perceived charisma on support ratings by .22 in comparison with the control condition. When we evaluate this difference in relation to the standard deviation of support ratings, we find that the upper bound has an effect size of *d*  = .17. Our data are consistent with mortality salience enhancing the difference between the two candidates with a small effect size, (*d* = .20), but inconsistent with medium and large effect sizes for the mortality salience by perceive charisma interaction.

**Appendix C – Regression Tables by Sample**

**Regression Tables by Sample**

Table 5. *Model Predicting Presidential Support for Sample 1*

| *Predictor* | *b* | *SE* | *Beta* | *T* | *p* |
| --- | --- | --- | --- | --- | --- |
| Intercept | 1.02 | .16 |  | 6.48 | .00 |
| Mortality Salience | -.10 | .16 | -.05 | -.60 | .55 |
| System Justification | -.24 | .11 | -.16 | -2.08 | .04 |
| Political Ideology | -.70 | .07 | -.74 | -9.36 | .00 |
| Mortality Salience * Ideology | -.11 | .07 | -.12 | -1.48 | .14 |
| System Justification * Ideology | -.13 | .05 | -.20 | -2.65 | .01 |
| Mortality Salience * System Justification | .29 | .11 | .19 | 2.59 | .01 |
| Mortality Salience * System Justification * Ideology | .08 | .05 | .12 | 1.57 | .12 |

Table 6. *Model Predicting Presidential Support for Sample 2*

| *Predictor* | *b* | *SE* | *Beta* | *T* | *p* |
| --- | --- | --- | --- | --- | --- |
| Intercept | 2.04 | .23 |  | 8.77 | .00 |
| Mortality Salience | -.12 | .28 | -.18 | -3.63 | .00 |
| System Justification | .40 | .19 | .27 | 2.09 | .04 |
| Political Ideology | -.52 | .10 | -.55 | -5.15 | .00 |
| Mortality Salience * Ideology | .12 | .10 | .13 | 1.22 | .22 |
| System Justification * Ideology | -.30 | .09 | -.45 | -3.46 | .00 |
| Mortality Salience * System Justification | -.24 | .19 | -.16 | -1.27 | .21 |
| Mortality Salience * System Justification * Ideology | .02 | .09 | .03 | .21 | .83 |

Table 7. *Model Predicting Presidential Support for Sample 3*

| *Predictor* | *b* | *SE* | *Beta* | *T* | *p* |
| --- | --- | --- | --- | --- | --- |
| Intercept | 1.02 | .11 |  | 9.35 | .00 |
| Mortality Salience | .04 | .11 | .02 | .35 | .73 |
| System Justification | -.04 | .09 | -.03 | -.51 | .61 |
| Political Ideology | -.69 | .05 | -.74 | -13.38 | .00 |
| Mortality Salience * Ideology | .09 | .05 | .10 | 1.81 | .07 |
| System Justification * Ideology | -.02 | .04 | -.03 | -.56 | .58 |
| Mortality Salience * System Justification | -.10 | .09 | -.07 | -1.14 | .26 |
| Mortality Salience * System Justification * Ideology | -.01 | .04 | -.02 | -.28 | .78 |

Table 8. *Model Predicting Presidential Support for Sample 4*

| *Predictor* | *b* | *SE* | *Beta* | *T* | *p* |
| --- | --- | --- | --- | --- | --- |
| Intercept | 1.03 | .10 |  | 10.58 | .00 |
| Mortality Salience | -.12 | .10 | -.06 | -1.25 | .21 |
| System Justification | -.20 | .07 | -.14 | -2.99 | .00 |
| Political Ideology | -.63 | .04 | -.68 | -14.43 | .00 |
| Mortality Salience * Ideology | .06 | .04 | .06 | 1.32 | .19 |
| System Justification * Ideology | -.05 | .03 | -.07 | -1.52 | .13 |
| Mortality Salience * System Justification | .15 | .07 | .10 | 2.19 | .03 |
| Mortality Salience * System Justification * Ideology | .04 | .03 | .06 | 1.31 | .19 |

*Note.* Political Ideology was measured with one item on a nine-point scale where higher numbers indicate greater conservatism. System justification was measured with eight items on a nine-point scale where higher numbers indicate stronger system justifying attitudes. Mortality salience was effect coded (-1 for the control condition, 1 for the mortality salience condition). Candidate support was measured with a difference score of Obama’s candidate support minus Romney’s candidate support (both measured with five items on a five-point scale where higher numbers indicate greater candidate support). Higher numbers on the difference score indicate exhibiting greater support towards Obama than towards Romney.

**Appendix D – Expanded model predicting support for candidates as a function of candidate identity (Obama vs. Romney), participant’s system justification and ideology scores, participants rating of candidate charisma, and whether the participant was in the mortality salience condition**

| *Predictor* | *b* | *SE* | *df* | *t* | *p* | *95% CI Lower* | *95% CI Upper* |
| --- | --- | --- | --- | --- | --- | --- | --- |
| Intercept | 2.81 | 0.04 | 866.72 | 66.45 | 0.00 | 2.73 | 2.89 |
| Candidate | -0.76 | 0.08 | 868.33 | -9.02 | 0.00 | -0.93 | -0.60 |
| SJ * Candidate | -0.07 | 0.06 | 930.10 | -1.05 | 0.29 | -0.20 | 0.06 |
| Ideology * Candidate | 0.49 | 0.04 | 910.60 | 13.84 | 0.00 | 0.42 | 0.56 |
| Ideology * SJ * Candidate | 0.13 | 0.03 | 947.45 | 4.27 | 0.00 | 0.07 | 0.19 |
| MortSal * Candidate | 0.19 | 0.17 | 883.63 | 1.11 | 0.27 | -0.14 | 0.51 |
| MortSal * SJ * Candidate | -0.13 | 0.13 | 954.21 | -1.01 | 0.31 | -0.39 | 0.12 |
| MortSal * Ideology * Candidate | -0.11 | 0.07 | 915.67 | -1.56 | 0.12 | -0.25 | 0.03 |
| MortSal * Charisma * Candidate | -0.12 | 0.14 | 769.08 | -0.81 | 0.42 | -0.40 | 0.17 |
| MortSal * Ideology * Charisma * Candidate | 0.09 | 0.06 | 849.05 | 1.54 | 0.12 | -0.02 | 0.20 |
| MortSal | 0.07 | 0.08 | 874.26 | 0.87 | 0.38 | -0.09 | 0.23 |
| Ideology | 0.02 | 0.02 | 911.56 | 1.24 | 0.22 | -0.01 | 0.06 |
| Charisma | 0.41 | 0.04 | 788.86 | 11.20 | 0.00 | 0.34 | 0.49 |
| SJ | 0.19 | 0.03 | 935.97 | 5.93 | 0.00 | 0.13 | 0.26 |
| MortSal * Ideology | 0.01 | 0.04 | 915.96 | 0.18 | 0.86 | -0.06 | 0.08 |
| MortSal * Charisma | 0.07 | 0.07 | 807.69 | 0.99 | 0.33 | -0.07 | 0.22 |
| MortSal * SJ | 0.01 | 0.06 | 916.06 | 0.11 | 0.91 | -0.11 | 0.13 |
| Ideology * Charisma | 0.02 | 0.01 | 847.90 | 1.20 | 0.23 | -0.01 | 0.05 |
| Ideology * SJ | -0.01 | 0.01 | 926.59 | -0.92 | 0.36 | -0.04 | 0.02 |
| Charisma * SJ | -0.06 | 0.03 | 944.05 | -2.12 | 0.04 | -0.11 | 0.00 |
| Charisma * Candidate | -0.20 | 0.07 | 769.43 | -2.71 | 0.01 | -0.34 | -0.05 |
| MortSal * Ideology * Charisma | -0.02 | 0.03 | 863.76 | -0.69 | 0.49 | -0.08 | 0.04 |
| MortSal * Ideology * SJ | -0.01 | 0.03 | 938.50 | -0.44 | 0.66 | -0.07 | 0.04 |
| MortSal * Charisma * SJ | -0.08 | 0.05 | 985.71 | -1.52 | 0.13 | -0.19 | 0.02 |
| Ideology * Charisma * SJ | 0.01 | 0.01 | 985.40 | 1.12 | 0.26 | -0.01 | 0.03 |
| Ideology * Charisma * Candidate | 0.13 | 0.03 | 841.15 | 4.46 | 0.00 | 0.07 | 0.18 |
| Charisma * SJ * Candidate | 0.13 | 0.05 | 819.98 | 2.69 | 0.01 | 0.03 | 0.22 |
| MortSal * Ideology * Charisma * SJ | 0.02 | 0.02 | 985.92 | 0.78 | 0.44 | -0.03 | 0.06 |
| MortSal * Ideology * SJ * Candidate | 0.01 | 0.06 | 956.50 | 0.24 | 0.81 | -0.10 | 0.13 |
| MortSal * Charisma * SJ * Candidate | -0.04 | 0.08 | 794.62 | -0.51 | 0.61 | -0.20 | 0.12 |
| Ideology * Charisma * SJ * Candidate | -0.01 | 0.02 | 844.14 | -0.48 | 0.63 | -0.04 | 0.02 |
| MortSal * Ideology * Charisma * SJ * Candidate | -0.01 | 0.04 | 918.48 | -0.37 | 0.71 | -0.09 | 0.06 |
| Sample 1 | -0.11 | 0.07 | 867.37 | -1.49 | 0.14 | -0.26 | 0.03 |
| Sample 2 | -0.01 | 0.10 | 857.51 | -0.06 | 0.95 | -0.20 | 0.19 |
| Sample 3 | 0.09 | 0.06 | 902.84 | 1.67 | 0.10 | -0.02 | 0.20 |
| Candidate * Sample 1 | 0.31 | 0.15 | 884.49 | 2.04 | 0.04 | 0.01 | 0.61 |
| Candidate * Sample 2 | -0.73 | 0.20 | 855.77 | -3.71 | 0.00 | -1.12 | -0.34 |
| Candidate * Sample 3 | 0.23 | 0.11 | 905.42 | 2.04 | 0.04 | 0.01 | 0.45 |
| SJ * Candidate * Sample 1 | 0.23 | 0.11 | 946.60 | 2.17 | 0.03 | 0.02 | 0.44 |
| SJ * Candidate * Sample 2 | -0.46 | 0.15 | 983.02 | -3.11 | 0.00 | -0.75 | -0.17 |
| SJ * Candidate * Sample 3 | 0.04 | 0.09 | 977.27 | 0.50 | 0.62 | -0.13 | 0.21 |
| Ideology * Candidate * Sample 1 | 0.02 | 0.06 | 880.47 | 0.31 | 0.76 | -0.10 | 0.14 |
| Ideology * Candidate * Sample 2 | -0.05 | 0.08 | 918.96 | -0.54 | 0.59 | -0.21 | 0.12 |
| Ideology * Candidate * Sample 3 | 0.01 | 0.05 | 909.46 | 0.15 | 0.88 | -0.09 | 0.10 |
| Ideology * SJ * Candidate * Sample 1 | -0.02 | 0.04 | 976.37 | -0.34 | 0.73 | -0.10 | 0.07 |
| Ideology * SJ * Candidate * Sample 2 | 0.26 | 0.07 | 982.03 | 3.42 | 0.00 | 0.11 | 0.40 |
| Ideology * SJ * Candidate * Sample 3 | -0.11 | 0.04 | 983.69 | -2.95 | 0.00 | -0.18 | -0.04 |
| MortSal * Candidate * Sample 1 | -0.56 | 0.30 | 872.85 | -1.89 | 0.06 | -1.14 | 0.02 |
| MortSal * Candidate * Sample 2 | 0.73 | 0.39 | 890.89 | 1.90 | 0.06 | -0.03 | 1.49 |
| MortSal * Candidate * Sample 3 | -0.19 | 0.22 | 909.72 | -0.87 | 0.38 | -0.62 | 0.24 |
| MortSal * SJ * Candidate * Sample 1 | -0.58 | 0.21 | 984.74 | -2.78 | 0.01 | -0.99 | -0.17 |
| MortSal * SJ * Candidate * Sample 2 | 0.41 | 0.29 | 973.55 | 1.42 | 0.16 | -0.16 | 0.98 |
| MortSal * SJ * Candidate * Sample 3 | 0.29 | 0.17 | 983.77 | 1.69 | 0.09 | -0.05 | 0.62 |
| MortSal * Ideology * Candidate * Sample 1 | 0.30 | 0.12 | 905.54 | 2.41 | 0.02 | 0.06 | 0.54 |
| MortSal * Ideology * Candidate * Sample 2 | -0.24 | 0.17 | 924.16 | -1.43 | 0.15 | -0.56 | 0.09 |
| MortSal * Ideology * Candidate * Sample 3 | -0.05 | 0.10 | 922.02 | -0.47 | 0.64 | -0.24 | 0.15 |
| MortSal * Charisma * Candidate * Sample 1 | 0.11 | 0.25 | 805.87 | 0.43 | 0.67 | -0.38 | 0.60 |
| MortSal * Charisma * Candidate * Sample 2 | -0.28 | 0.34 | 729.69 | -0.83 | 0.41 | -0.94 | 0.38 |
| MortSal * Charisma * Candidate * Sample 3 | 0.20 | 0.19 | 830.82 | 1.10 | 0.27 | -0.16 | 0.57 |
| MortSal * Ideology * Charisma * Candidate * Sample 1 | -0.07 | 0.09 | 876.22 | -0.78 | 0.44 | -0.25 | 0.11 |
| MortSal * Ideology * Charisma * Candidate * Sample 2 | 0.18 | 0.14 | 826.39 | 1.25 | 0.21 | -0.10 | 0.45 |
| MortSal * Ideology * Charisma * Candidate * Sample 3 | -0.09 | 0.08 | 882.32 | -1.17 | 0.24 | -0.24 | 0.06 |
| MortSal * Sample 1 | 0.08 | 0.15 | 877.88 | 0.55 | 0.58 | -0.21 | 0.37 |
| MortSal * Sample 2 | -0.03 | 0.19 | 875.18 | -0.17 | 0.87 | -0.41 | 0.34 |
| MortSal * Sample 3 | 0.05 | 0.11 | 911.43 | 0.44 | 0.66 | -0.17 | 0.27 |
| Ideology * Sample 1 | 0.00 | 0.03 | 871.72 | 0.08 | 0.94 | -0.06 | 0.06 |
| Ideology * Sample 2 | 0.03 | 0.04 | 923.74 | 0.63 | 0.53 | -0.06 | 0.11 |
| Ideology * Sample 3 | 0.01 | 0.02 | 912.03 | 0.21 | 0.83 | -0.04 | 0.05 |
| Charisma * Sample 1 | 0.05 | 0.06 | 815.28 | 0.73 | 0.47 | -0.08 | 0.17 |
| Charisma * Sample 2 | -0.07 | 0.09 | 767.52 | -0.79 | 0.43 | -0.25 | 0.11 |
| Charisma * Sample 3 | -0.01 | 0.05 | 839.49 | -0.24 | 0.81 | -0.10 | 0.08 |
| SJ * Sample 1 | 0.07 | 0.05 | 942.86 | 1.26 | 0.21 | -0.04 | 0.17 |
| SJ * Sample 2 | 0.05 | 0.07 | 983.87 | 0.65 | 0.52 | -0.10 | 0.19 |
| SJ * Sample 3 | -0.08 | 0.04 | 962.07 | -1.82 | 0.07 | -0.16 | 0.01 |
| MortSal * Ideology * Sample 1 | -0.07 | 0.06 | 897.54 | -1.11 | 0.27 | -0.19 | 0.05 |
| MortSal * Ideology * Sample 2 | 0.04 | 0.08 | 926.07 | 0.44 | 0.66 | -0.13 | 0.20 |
| MortSal * Ideology * Sample 3 | -0.01 | 0.05 | 917.29 | -0.30 | 0.77 | -0.11 | 0.08 |
| MortSal * Charisma * Sample 1 | -0.25 | 0.13 | 817.13 | -1.96 | 0.05 | -0.49 | 0.00 |
| MortSal * Charisma * Sample 2 | 0.20 | 0.18 | 803.34 | 1.15 | 0.25 | -0.14 | 0.55 |
| MortSal * Charisma * Sample 3 | -0.02 | 0.10 | 859.08 | -0.19 | 0.85 | -0.20 | 0.17 |
| MortSal * SJ * Sample 1 | 0.02 | 0.09 | 961.61 | 0.23 | 0.82 | -0.15 | 0.19 |
| MortSal * SJ * Sample 2 | -0.09 | 0.13 | 984.36 | -0.74 | 0.46 | -0.34 | 0.15 |
| MortSal * SJ * Sample 3 | 0.07 | 0.07 | 980.76 | 0.99 | 0.32 | -0.07 | 0.22 |
| Ideology * Charisma * Sample 1 | -0.01 | 0.02 | 858.67 | -0.22 | 0.83 | -0.05 | 0.04 |
| Ideology * Charisma * Sample 2 | 0.01 | 0.04 | 826.27 | 0.41 | 0.68 | -0.06 | 0.09 |
| Ideology * Charisma * Sample 3 | -0.02 | 0.02 | 878.61 | -1.15 | 0.25 | -0.06 | 0.02 |
| Ideology * SJ * Sample 1 | -0.02 | 0.02 | 963.29 | -0.80 | 0.43 | -0.06 | 0.02 |
| Ideology * SJ * Sample 2 | -0.02 | 0.03 | 971.02 | -0.72 | 0.48 | -0.09 | 0.04 |
| Ideology * SJ * Sample 3 | 0.04 | 0.02 | 977.26 | 2.17 | 0.03 | 0.00 | 0.07 |
| Charisma * SJ * Sample 1 | 0.02 | 0.05 | 877.02 | 0.41 | 0.68 | -0.07 | 0.11 |
| Charisma * SJ * Sample 2 | -0.08 | 0.07 | 986.01 | -1.18 | 0.24 | -0.21 | 0.05 |
| Charisma * SJ * Sample 3 | 0.01 | 0.04 | 930.96 | 0.20 | 0.84 | -0.06 | 0.08 |
| Charisma * Candidate * Sample 1 | -0.13 | 0.12 | 800.21 | -1.01 | 0.31 | -0.37 | 0.12 |
| Charisma * Candidate * Sample 2 | -0.12 | 0.17 | 732.27 | -0.68 | 0.50 | -0.46 | 0.22 |
| Charisma * Candidate * Sample 3 | 0.18 | 0.09 | 835.08 | 1.86 | 0.06 | -0.01 | 0.36 |
| MortSal * Ideology * Charisma * Sample 1 | 0.05 | 0.05 | 881.47 | 1.12 | 0.26 | -0.04 | 0.14 |
| MortSal * Ideology * Charisma * Sample 2 | -0.10 | 0.07 | 852.47 | -1.35 | 0.18 | -0.24 | 0.04 |
| MortSal * Ideology * Charisma * Sample 3 | 0.04 | 0.04 | 890.20 | 0.92 | 0.36 | -0.04 | 0.11 |
| MortSal * Ideology * SJ * Sample 1 | -0.05 | 0.04 | 967.59 | -1.19 | 0.24 | -0.13 | 0.03 |
| MortSal * Ideology * SJ * Sample 2 | 0.02 | 0.06 | 981.75 | 0.37 | 0.71 | -0.10 | 0.15 |
| MortSal * Ideology * SJ * Sample 3 | 0.03 | 0.03 | 937.56 | 0.84 | 0.40 | -0.04 | 0.10 |
| MortSal * Charisma * SJ * Sample 1 | -0.14 | 0.09 | 804.82 | -1.60 | 0.11 | -0.31 | 0.03 |
| MortSal * Charisma * SJ * Sample 2 | 0.01 | 0.12 | 714.63 | 0.06 | 0.95 | -0.23 | 0.24 |
| MortSal * Charisma * SJ * Sample 3 | 0.08 | 0.07 | 812.24 | 1.23 | 0.22 | -0.05 | 0.22 |
| Ideology * Charisma * SJ * Sample 1 | -0.01 | 0.02 | 862.78 | -0.83 | 0.41 | -0.05 | 0.02 |
| Ideology * Charisma * SJ * Sample 2 | 0.06 | 0.03 | 854.71 | 2.39 | 0.02 | 0.01 | 0.12 |
| Ideology * Charisma * SJ * Sample 3 | -0.03 | 0.01 | 817.60 | -1.99 | 0.05 | -0.06 | 0.00 |
| Ideology * Charisma * Candidate * Sample 1 | 0.00 | 0.05 | 853.45 | 0.03 | 0.98 | -0.09 | 0.09 |
| Ideology * Charisma * Candidate * Sample 2 | 0.07 | 0.07 | 816.67 | 0.93 | 0.35 | -0.07 | 0.21 |
| Ideology * Charisma * Candidate * Sample 3 | -0.02 | 0.04 | 874.12 | -0.60 | 0.55 | -0.10 | 0.05 |
| Charisma * SJ * Candidate * Sample 1 | 0.11 | 0.09 | 855.25 | 1.18 | 0.24 | -0.07 | 0.28 |
| Charisma * SJ * Candidate * Sample 2 | 0.08 | 0.10 | 900.07 | 0.84 | 0.40 | -0.11 | 0.28 |
| Charisma * SJ * Candidate * Sample 3 | -0.16 | 0.07 | 890.88 | -2.43 | 0.02 | -0.30 | -0.03 |
| MortSal * Ideology * Charisma * SJ * Sample 1 | 0.01 | 0.04 | 961.39 | 0.28 | 0.78 | -0.06 | 0.08 |
| MortSal * Ideology * Charisma * SJ * Sample 2 | 0.01 | 0.05 | 920.57 | 0.17 | 0.86 | -0.09 | 0.11 |
| MortSal * Ideology * Charisma * SJ * Sample 3 | -0.01 | 0.03 | 959.53 | -0.48 | 0.63 | -0.07 | 0.04 |
| MortSal * Ideology * SJ * Candidate * Sample 1 | 0.00 | 0.09 | 984.73 | -0.01 | 0.99 | -0.17 | 0.17 |
| MortSal * Ideology * SJ * Candidate * Sample 2 | 0.05 | 0.14 | 985.52 | 0.37 | 0.71 | -0.22 | 0.33 |
| MortSal * Ideology * SJ * Candidate * Sample 3 | 0.01 | 0.08 | 976.20 | 0.10 | 0.92 | -0.14 | 0.16 |

*Note.* Political Ideology was measured with one item on a nine-point scale where higher numbers indicate greater conservatism. System justification was measured with eight items on a nine-point scale where higher numbers indicate stronger system justifying attitudes. Charisma was measured with one item on a five-point scale where higher numbers indicate greater charisma. Mortality salience was effect coded (-0.5 for the control condition, .5 for the mortality salience condition). Candidate was effect coded (-0.5 for Obama, .5 for Romney). Sample was effect coded with Sample 4 serving as the reference group. Candidate support was measured with a difference score of Obama’s candidate support minus Romney’s candidate support (both measured with five items on a five-point scale where higher numbers indicate greater candidate support). Higher numbers on the difference score indicate exhibiting greater support towards Obama than towards Romney.
